# Phase-Controlled Iron Oxide Nanobox Deposited on Hierarchically Structured Graphene Networks for Lithium Ion Storage and Photocatalysis

**DOI:** 10.1038/srep19959

**Published:** 2016-01-29

**Authors:** Sol Yun, Young-Chul Lee, Ho Seok Park

**Affiliations:** 1 School of Chemical Engineering, Sungkyunkwan University, Suwon, 16419, Republic of Korea; 2 Department of BioNano Technology, Gachon University, Seongnam, 13120, Republic of Korea

## Abstract

The phase control, hierarchical architecturing and hybridization of iron oxide is important for achieving multifunctional capability for many practical applications. Herein, hierarchically structured reduced graphene oxide (hrGO)/*α*-Fe_2_O_3_ and *γ*-Fe_3_O_4_ nanobox hybrids (hrGO/*α*-Fe and hrGO/*γ*-Fe NBhs) are synthesized via a one-pot, hydrothermal process and their functionality controlled by the crystalline phases is adapted for energy storage and photocatalysis. The three-dimensionally (3D) macroporous structure of hrGO/*α*-Fe NBhs is constructed, while *α*-Fe_2_O_3_ nanoboxes (NBs) in a proximate contact with the hrGO surface are simultaneously grown during a hydrothermal treatment. The discrete *α*-Fe_2_O_3_ NBs are uniformly distributed on the surface of the hrGO/*α*-Fe and confined in the 3D architecture, thereby inhibiting the restacking of rGO. After the subsequent phase transition into *γ*-Fe_3_O_4_, the hierarchical structure and the uniform distribution of NBs are preserved. Despite lower initial capacity, the hrGO/*α*-Fe NBhs show better rate and cyclic performances than those of commercial rGO/*α*-Fe due to the uniform distribution of discrete *α*-Fe_2_O_3_ NBs and electronic conductivity, macroporosity, and buffering effect of the hrGO for lithium ion battery anodes. Moreover, the catalytic activity and kinetics of hrGO/*γ*-Fe NBhs are enhanced for photo-Fenton reaction because of the uniform distribution of discrete *γ*-Fe_3_O_4_ NBs on the 3D hierarchical architecture.

Iron oxide nanoparticles (NPs) are considered as an attractive energy storage and catalyst material due to high theoretical lithium capacity, good (electro)catalytic activity, environmental friendliness, high abundance, and low cost^1^,^2^. In order to manipulate physical and electrochemical properties, diverse nanostructures of iron oxide NPs such as porous particle^3^, 0D nanoparticle^4^,^5^, 1D nanowires and nanotubes^6^,^7^,^8^, 2D nanoflakes and nanosheet^9^,^10^, and 3D flowerlike and hollow structures^11^,^12^ have been developed for applications into lithium ion battery (LIB) anode and photocatalysis. Along with the nanoscale architecturing, the crystalline phases (e.g. hematite, maghemite and magnetite) and oxidation states (e.g. Fe^2+^ and Fe^3+^) of iron oxide need to be delicately controlled for achieving desirable properties and for adapting functionality^13^. Despite these features originating from structure and crystalline phase, iron oxide NP itself as a single component has its own limitations. For instance, iron oxide NPs obtain the shortcomings of low electronic conductivity and electrode pulverization when they electrochemically store lithium ions^1^, thereby revealing limited rate and cyclic performances. Furthermore, photo-Fenton reaction using iron oxides, which is very useful for the degradation or mineralization of harmful/toxic pollutants in water and wastewater, demonstrated the drawbacks of poor recycling and low catalyst stability^6^. Accordingly, much effort has been devoted to resolve the afore-mentioned challenges by nanostructuring and hybridization^1^,^13^.

Recently, the integration of metal oxide (MO) active materials with the conductive carbon matrices has gained significant attention as an emerging class of functional hybrid materials owing to the appealing electrochemical properties, large surface area, and chemical and mechanical stabilities of carbon nanomaterials for energy storage and catalytic applications^14^,^15^. Various iron oxide/carbon nanocomposites such as 1D iron oxide/carbon nanowires^16^, 2D iron oxide/carbon hybrid nanosheets^17^, and 3D iron oxide/carbon nanostructures^18^ have been developed for improving the electrochemical and catalytic properties of iron oxide. In particular, hierarchical architecturing of graphene/MO hybrids in a three-dimensional (3D) manner is expected to become an innovative chemical approach for full potential of respective functionality. In addition to intrinsic material properties, such a hierarchical structure constructed by graphene nanosheets and MO NPs takes advantages of 3D interconnected macroscopic structure in terms of a large accessible area, fast mass and ion transport, percolated charge transfer, and structural integrity^19^,^20^,^21^. For instance, Ma *et al.* demonstrated one-step, solvothermal synthesis of 3D self-assembled reduced graphene oxide (rGO)/CoO hybrids^22^. Wu *et al.* reported 3D graphene aerogel/Fe_3_O_4_ hybrids for electrocatalysis^23^. However, neither the synthetic method was environmentally benign nor multi-functionality of as-obtained hybrid was demonstrated. Accordingly, it is important to develop the one-pot synthesis of hierarchically structured graphene/MO nanohybrids through solution chemistry and subsequent phase control of iron oxide NPs for specific applications.

Herein, we demonstrate the one-pot, hydrothermal synthesis of hierarchically structured rGO/*α*-Fe_2_O_3_ and rGO/*γ*-Fe_3_O_4_ nanobox hybrids (hrGO/*α*-Fe and hrGO/*γ*-Fe NBhs) for LIB anodes and photo-Fenton catalysis. All in one synthetic approach is very simple yet useful for simultaneously constructing 3D macroscopic rGO structures and growing *α*-Fe_2_O_3_ NBs. Even after the subsequent reduction from *α*-Fe_2_O_3_ into *γ*-Fe_3_O_4_, hierarchical architectures remained intact. These hrGO/Fe NBhs showed excellent battery and photocatalytic performances.

## Results

### Structure of the hrGO/*α*-Fe NBhs

**Figure 1:**
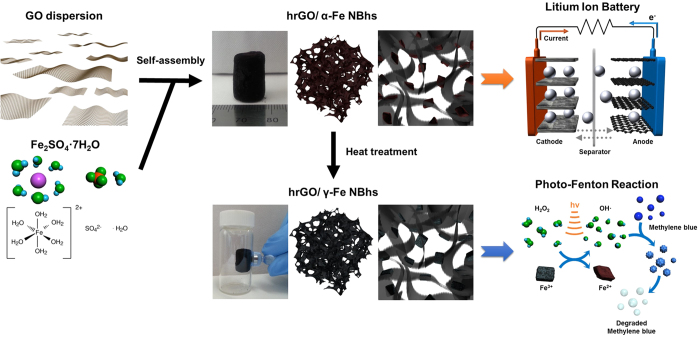
Synthetic procedures of hrGO/Fe NBhs through a one-pot, hydrothermal process and application into LIB anodes and photo-Fenton catalysis.

As illustrated in Fig. 1, the *α*-Fe_2_O_3_ NBs supported onto the surface of 3D hierarchically structured rGO frameworks were synthesized through a one-pot, hydrothermal method. First, GOs obtained from the modified Hummers’ method were dispersed in DI water. The iron precursor of FeSO_4_ ·7H_2_ O was added to aqueous GO dispersions at the fixed concentration of 0.1 mmol/mL. The 3D internetworked macroporous structures could not be constructed at a higher precursor concentration, while the loading of *α*-Fe_2_O_3_ became smaller at a lower precursor concentration. The resulting mixture was a uniform and opaque dispersion due to the role of GO as a surfactant, which is very crucial for the formation of 3D internetworked structure through a self-assembly. The oxygen functional groups on the surfaces of the GO sheets interact with Fe^3+^ ions through an electrostatic attraction and then, act as anchoring *α*-Fe_2_O_3_ NBs during a NP growth in a similar manner to functionalized carbon nanotube and graphene as previously demonstrated by us^24^,^25^. The organization and assembly of GO colloids into a 3D hierarchically structured rGO hydrogels was driven by *π*-*π* stacking interactions of conjugated structure during a hydrothermal reaction at 180 °C for 12 h. The formation of *α*-Fe_2_O_3_ NBs was driven by nucleation and growth steps during a hydrothermal process. In other words, Fe^3+^ ions adsorbed on GO sheets react to form nanocrystalline *α*-Fe_2_O_3_ NBs through a hydrolysis and condensation at high temperatures and pressures. Simultaneously, GOs interacting with Fe precursors are reduced and self-assembled to construct the 3D macroporous rGO networks. The resulting product was obtained in a form of a red hydrogel of hrGO/*α*-Fe NBhs, which was further treated by several washing to remove impurities and freeze drying to preserve 3D macroporous monoliths. Finally, the phase of iron oxide supported on the hrGO macrostructures was changed into *γ*-Fe_3_O_4_ after a heat treatment under a reducing atmosphere of H_2_/N_2_ mixture. As demonstrated by magnetic property of *γ*-Fe_3_O_4_, the hrGO/*γ*-Fe NBhs are very useful for catalytic applications due to the feasibility of catalyst separation by magnetic force.

**Figure 2:**
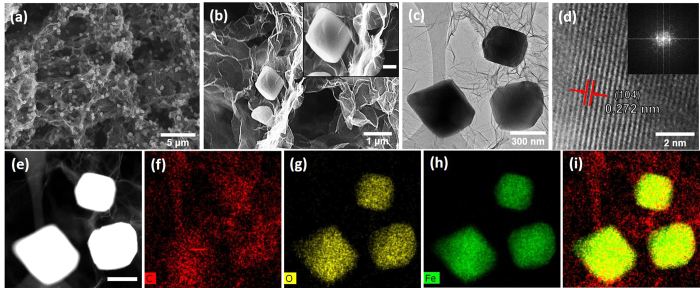
(**a**) Low and (**b**) high magnificent SEM (inset; scale bar, 300 nm), (**c**) and (**d**) TEM, and (**e**) Dark-field TEM and (**f–i**) STEM images of hrGO/*α*-Fe NBhs. Inset of (**d**) is corresponding SAED pattern.

The morphologies of hrGO/*α*-Fe NBhs were investigated by scanning electron microscopy (SEM), transmission electronmicroscopy (TEM), and scanning TEM (STEM) as shown in Fig. 2. The hrGO/*α*-Fe NBhs revealed the 3D macroporous networks, where the pore walls were composed of stacked rGO sheets, in an analogous manner to hrGO (see Fig. S1), as observed by SEM image. This observation indicates that the formation of 3D hierarchical structure was not hindered by the growth of *α*-Fe_2_O_3_ NBs. Accordingly, the 3D interconnected macropores of the hrGO/*α*-Fe NBhs were mainly attributed to the assembly of Fe precursor-supported, few-layered rGO sheets. On the other hand, the aggregation of irregular Fe_2_O_3_ particles was observed when they were synthesized without the rGO under the same synthetic condition as the hrGO/*α*-Fe NBhs (see Fig. S2). This result was attributed to the role of rGO on the regulation of *α*-Fe_2_O_3_ NB growth and the deposition of discrete Fe_2_O_3_ NBs. Compared to the macropore size of the hrGO, that of hrGO/*α*-Fe NBhs was slightly smaller due to the different self-assembly chemistries arising from the existence of *α*-Fe_2_O_3_ NBs. Nonetheless, the 3D macroporous structure of the hrGO and the uniform distribution of *α*-Fe_2_O_3_ NBs were achieved for the hrGO/*α*-Fe NBhs. The open macroporous continuity facilitates ion and chemical diffusion to electrochemically or catalytic active sites of iron oxides with less charge and mass transport resistance. For the hrGO/*α*-Fe NBhs, the particle size of *α*-Fe_2_O_3_ NBs ranges from 500 to 600 nm as shown in a high magnificent SEM image. Moreover, *α*-Fe_2_O_3_ NBs are in a very proximate contact with the rGO layers, indicating that they are grown and confined onto the hrGO surface. The existence and uniform distribution of *α*-Fe_2_O_3_ NBs deposited on the hrGO surfaces was further confirmed by elementary mappings and overlay of C, O and Fe signals in STEM images. The contrast difference between rGO and *α*-Fe_2_O_3_ NBs was clearly observed by the dark field TEM images, again verifying the existence and distribution of *α*-Fe_2_O_3_ NPs. This uniform distribution and proximate contact of *α*-Fe_2_O_3_ NBs onto the surface of hrGO would maximize the effect of hybrid composition on the electrochemical and catalytic performances.

The crystalline structure of hrGO/*α*-Fe NBhs was characterized by the selected area diffraction (SAED) pattern, the HR-TEM image, and XRD pattern. As demonstrated by a HR-TEM image, the lattice resolved fringes of *α*-Fe_2_O_3_ NBs with constant spacing of 0.272 nm are indicative of the formation of crystalline structure during a one-pot, hydrothermal process. The crystalline structure of *α*-Fe_2_O_3_ NBs was further confirmed by XRD spectra. The major XRD pattern of hrGO/*α*-Fe NBhs was indexed to the characteristic peaks of rhombohedral phase (JCPDS 33-0664) of *α*-Fe_2_O_3_. In particular, the interplanar spacing of 0.272 nm observed by a HR-TEM image corresponded to the intense XRD peak of (104) plane. No obvious characteristic peak of the rGO was observed in the hrGO/*α*-Fe NBhs, suggesting that the restacking of graphene layers along a z-axis was prohibited by the confinement of *α*-Fe_2_O_3_ NBs onto the rGO layers. Therefore, the hierarchical structure of the hrGO/*α*-Fe NBhs and the formation and uniform distribution of the *α*-Fe_2_O_3_ NBs were readily achieved via a one-pot, hydrothermal assembly.

### Chemistry and textural property of the hrGO/*α*-Fe NBhs

**Figure 3:**
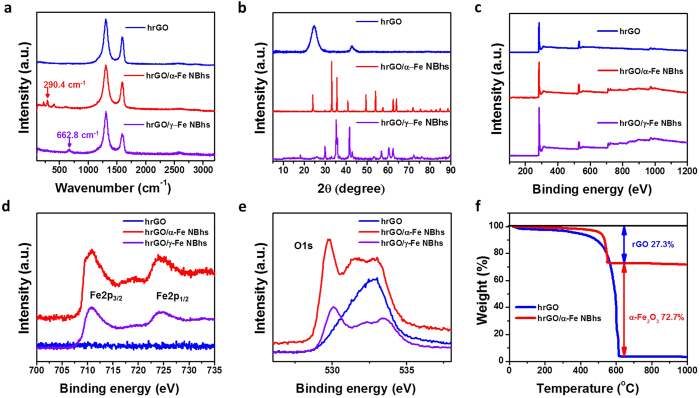
(**a**) XRD, (**b**) Raman, and (**c**) full scan, (**d**) Fe2p, and (**e**) O1s XPS spectra of hrGO, hrGO/*α*-Fe NBhs and hrGO/*γ*-Fe NBhs. (**f**) TGA of hrGO, and hrGO/*α*-Fe NBhs.

The chemical structure and composition of hrGO/*α*-Fe NBhs were investigated by Raman, TGA, and XPS analyses as shown in Fig. 3. The characteristic bands for carbon nanomaterials are identified by the disordered D and graphitic G bands at about 1350 and about 1590 cm^*−*1^, respectively. The distinct D bands of hrGO and hrGO/*α*-Fe NBhs indicate the disordered structure originating from numerous dangling bonds and defects. The I_*D*_/I_*G*_ of hrGO/*α*-Fe NBhs was comparable to that of hrGO, meaning that the formation and direct deposition of *α*-Fe_2_O_3_ NBs did not significantly destruct the conjugated structure of the hrGO surfaces in the course of hydrothermal reaction. The loading amount of *α*-Fe_2_O_3_ NBs were evaluated by TGA analysis. The thermal decomposition of hrGO occurred at around 500 °C as shown in TGA curve of pristine hrGO. This decomposition temperature was lowered for hrGO/*α*-Fe NBhs due to the catalytic effect of *α*-Fe_2_O_3_. The composition of hrGO/*α*-Fe NBhs was determined to be 72.7 and 27.3 wt% of *α*-Fe_2_O_3_ and hrGO, respectively.

The chemical identity and circumstance of *α*-Fe_2_O_3_ NBs in hybrids can be confirmed by XPS spectra. The full XPS spectrum revealed the presence of only the elements C, O, and Fe in the hybrid. We observed four different peaks of C1s, corresponding to sp2 (Cg, 285 eV), epoxy/hydroxyl groups (C–O, 286.5 eV), carbonyl groups (C=O, 287.8 eV), and carboxyl groups (O–C=O, 289.5 eV), respectively. The fraction of sp2 and C/O ratio of the hrGO/*α*-Fe NBhs was nearly identical to those of the hrGO. This finding supports no significant destruction of conjugated structure by *α*-Fe_2_O_3_ NBs as verified by Raman spectra. The high-resolution Fe2p peaks of the hrGO/*α*-Fe NBhs were observed at 710.6 and 724.2 eV with a 13.6 eV peak-to-peak separation, corresponding to the binding energy of Fe2p_3/2_ and Fe2p_1/2_ of *α*-Fe_2_O_3_ NBs, respectively. This finding implies the presence of Fe(III) which is in a good agreement with the valence of *α*-Fe_2_O_3_ phase as demonstrated by XRD result. Therefore, XRD, Raman, TGA, and XPS characterizations confirmed the identity and composition of crystalline *α*-Fe_2_O_3_ NBs in hybrids.

We measured the textural properties of the hrGO and hrGO/*α*-Fe NBhs using N_2_ adsorption/desorption isotherms (see Fig. S3). The mesopores of the hrGO/*α*-Fe NBhs with the size of 15.9 nm originating from the intervoids of rGO sheets were clearly observed by BJH analysis with the surface area of 60.5 m^2^/g and the pore volume of 0.240 cm^3^/g. The specific surface area and pore volume of the hrGO/*α*-Fe NBhs were lower compared to those of the hrGO by means of the mesopore blocking by *α*-Fe_2_O_3_ NBs. It was confirmed by the fact that the hrGO/*α*-Fe NBhs exhibited the disappearance of typical type-IV isotherm behavior, which was observed by the hrGO, corresponding to the existence of mesopores.

### Reduction from *α*-Fe_2_O_3_ into*γ*-Fe_3_O_4_ Nanobox

**Figure 4:**
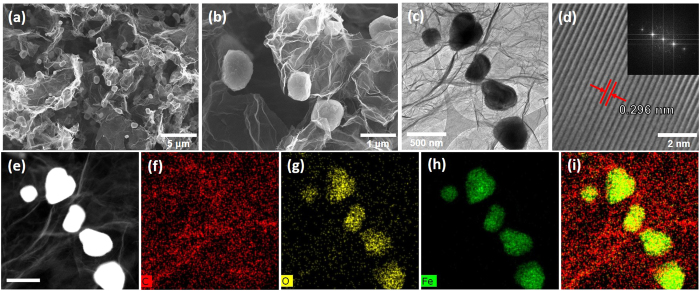
(**a**) Low and (**b**) high magnificent SEM, (**c**) and (**d**) TEM, and (**e**) Dark-field TEM and (**f–i**) STEM images of hrGO/*γ*-Fe NBhs. Inset of (**d**) is corresponding SAED pattern.

Since the physical and chemical properties of iron oxide depend on the types of phases, we tried to change *α*-phase into a reduced form of *γ*-phase. The hrGO/*α*-Fe NBhs were thermally treated at 500 °C under a reducing environment (of a mixed gas of 5/95 H_2_/N_2_) varying reduction period from 15 minutes to 2 hours. The morphology and composition of the hrGO/*γ*-Fe NBhs were characterized as shown in Fig. 4. As shown in optical images, the hrGO/*α*-Fe NBhs became a little shrunk because of the oxygen removal and magnetically active due to the phase transformation into *γ*-Fe_3_O_4_ phase. Compared to the morphology of *α*-Fe_2_O_3_ NPs before the reduction, the edges of *γ*-Fe_3_O_4_ NPs shaved away and they were slightly elongated during a thermal treatment. The change in the crystalline phase after the reduction was observed by XRD spectra. The discernible peaks can be indexed to (220), (311), (222), (400), (422), (511), (440), and (531) planes, reflecting the spinel structure of magnetite of *γ*-Fe_3_O_4_ (JCPDS No. 19-0629). A diffraction hump was seen in the range from 24 to 28°, which originates from hrGO (Fig. 3b). The lattice fringe of *γ*-Fe_3_O_4_ with a constant spacing of 0.296 nm was associated with the intense XRD peak of (220) plane. As shown by Raman spectra, the distinct peaks of hrGO/*γ*-Fe NBhs at 662.8 cm^*−*1^, which were different from the characteristic peaks of *α*-Fe_2_O_3_ at 290.4 cm^*−*1^, represented the successful reduction of *α*-Fe_2_O_3_ into *γ*-Fe_3_O_4_. Furthermore, the oxidation state of iron oxide was changed from Fe(III) into Fe(II) after thermal reduction as confirmed by XPS analysis. Along with the successful phase transition into *γ*-Fe_3_O_4_, the hierarchical morphology of hrGO/*α*-Fe NBhs was preserved. The existence and uniform distribution of *γ*-Fe_3_O_4_ deposited on the hrGO surface was confirmed by C, O and Fe signal as shown in STEM images. These results indicate that the *γ*-Fe_3_O_4_ NBs were successfully obtained by reducing the hrGO/*α*-Fe NBhs while maintaining the hierarchical architectures. Such a phase transition of iron oxide is very important for catalytic activity because the functionality of iron oxide depends on the crystalline structure and oxidation state of Fe.

### Applications of the hrGO/Fe NBhs into energy storage and photocatalysis

**Figure 5:**
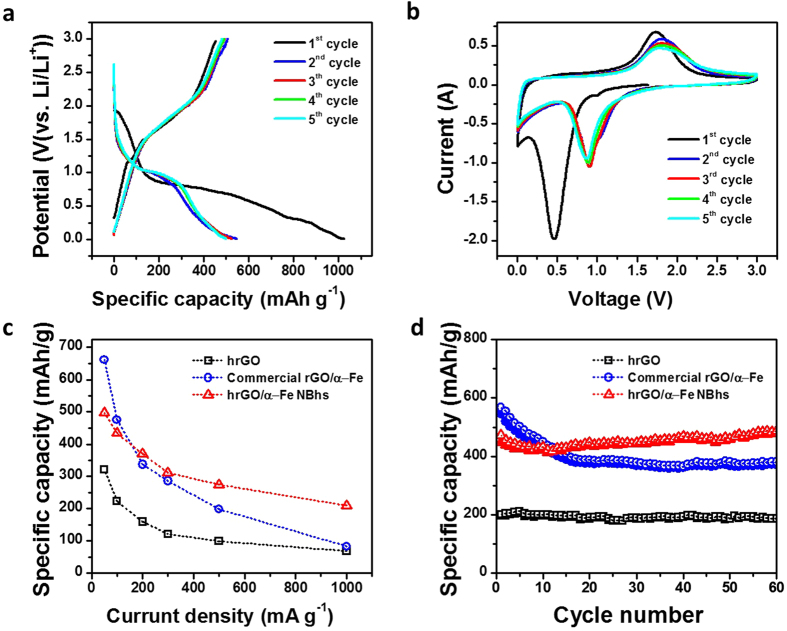
Galvanostatic charge and discharge voltage profiles of (a) hrGO/*α*-Fe NBhs at a current density of 50 mA/g. (b) CV curves of hrGO/*α*-Fe NBhs for the first 5 cycles at a scan rate of 0.5 mV/s. Comparison of (c) rate capability and (d) cycling performance of hrGO, commercial rGO/*α*-Fe and hrGO/*α*-Fe NBhs at a current density of 100 mA/g.

In order to demonstrate the superiority of the hrGO/Fe NBhs, we applied them into LIB anodes and photo-Fenton catalysis. The anode performances of hrGO/*α*-Fe NBhs were compared with those of the commercial rGO/*α*-Fe and hrGO as shown in Fig. 5 (see Figs S4, S5). We controlled the same composition of the commercial rGO/*α*-Fe, where the rGO powder was physically mixed with the commercial *α*-Fe_2_O_3_ NPs, as the hrGO/*α*-Fe NBhs to investigate the effect of 3D hierarchical structure and *α*-Fe_2_O_3_ NBs on the LIB anode performance. The GCD curves were measured at 50, 100, 200, 300, 500, and 1000 mA/g in the range of cut-off voltag from 0.01 V to 3.0 V (Fig. 5a), which is a typical operation range of *α*-Fe_2_O_3_^26^. The hrGO showed a sloped shape of GCD curve arising from the double layer capacitive behavior. By contrast, both commercial rGO/*α*-Fe and hrGO/*α*-Fe NBhs revealed a plateau region at 0.9 V corresponding to the conversion reaction of Fe(II) to Fe(0) through the following redox reaction; *α*-Fe_2_O_3_ + 6Li^+^ + 6e^*−*^*↔* 2Fe + 3Li_2_ O. This overall reaction can be divided into the individual reaction pathways^27^,^28^.


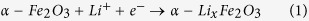



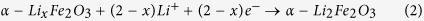



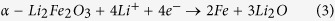


The irreversible phase transition of reaction (1) and (2) and solid electrolyte interphase (SEI) layer formation was associated with cathodic peak at 0.5 V as shown in the CV curve of the hrGO/*α*-Fe NBhs. As shown in CV curves, the initial five cycles of hrGO/*α*-Fe NBhs were obtained from 0.01 V to 3.0 V at scan rate of 0.5 mV/s (Fig. 5b). At the 1^*st*^cycle, the profound cathodic peak at 0.5 V corresponds to the formation of SEI and the complete reduction to Fe(0), as previously observed by the literature^29^. As a result of the irreversible capacity loss of *α*-Fe_2_O_3_, both of commercial rGO/*α*-Fe and hrGO/*α*-Fe NBhs reveal the low coulombic efficiencies of 48.0% (from 1701.3 to 816.1 mAh/g) and 44.6% (from 1026.2 to 457.9 mAh/g) at the 1^*st*^cycle, respectively, which was commonly demonstrated by the various nanostructured metal oxide anodes^27^,^30^,^31^,^32^. The reversible transition from Fe(0) to Fe(II) happens at a broad peak at around 1.7 V. From the 2^*nd*^cycle, the cathodic peak at 0.5 V disappears and new peak appears at 0.9 V, while the anodic peak is slightly shifted to 1.8 V. A pair of peaks at 1.8 and 0.9 V from the 2^*nd*^cycles, which is in a good agreement with the plateau voltages in the GCD curves, corresponds to the formation of cubic Li_2_ Fe_2_O_3_ phase and the reversible conversion reaction between Fe(II) and Fe(0) as previously observed in *α*-Fe_2_O_3_. Such a reversible transition was associated with the high coulombic efficiencies (>90%) of the hrGO/*α*-Fe NBhs from the 2^*nd*^cycles.

In order to understand the effect of the hierarchical architecture on the battery performances, the rate capability (Fig. 5c) and cyclic stability (Fig. 5d) of three samples were compared. The specific capacity of the commercial rGO/*α*-Fe dramatically decreased from 662.6 mAh/g at 50 mA/g to 83.6 mAh/g at 1000 mA/g with the capacity retention of 12.6%. In a sharp contrast to the commercial rGO/*α*-Fe, the hrGO/*α*-Fe NBhs exhibited better rate performance from 497.7 mAh/g to 210.3 mAh/g with the capacity retention of 42.3%. After 60 cycles at 100 mA/g, the commercial rGO/*α*-Fe showed highest initial discharge capacity of 566.5 mAh/g, but it was further decreased to 380.8 mAh/g (67.2%) of initial capacity. By contrast, the hrGO/*α*-Fe NBhs showed no capacity fading, maintaining initial capacity of 472.8 mAh/g. Apparently, the hrGO/*α*-Fe NBhs demonstrates a much better cyclic retention of 102.7% than that of 67.2% for the commercial rGO/*α*-Fe due to the 3D hierarchical structure. For comparison, the specific capacity of all the electrodes based on the total mass has also been calculated. And the details are mentioned in supporting information (see Table. S1).

**Figure 6:**
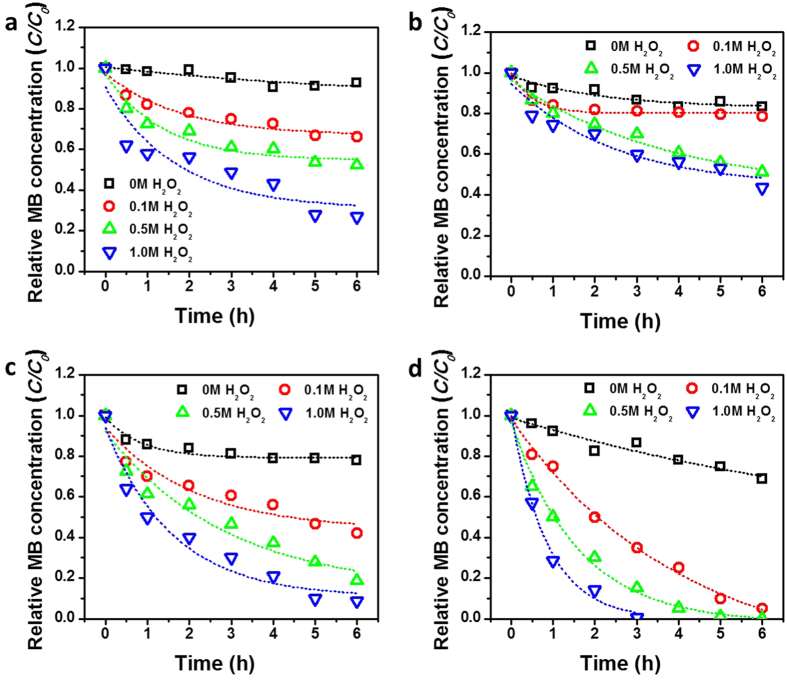
Photo-Fenton reaction. (**a**) MB degradation by photolysis without catalysts, (**b**) 15 min-, (**c**) 1 h-, and (**d**) 2 h-H_2_ hrGO/*γ*-Fe NBhs according to H_2_O_2_ concentrations (0, 0.1, 0.5, and 1.0 M) under 365 nm UV irradiation for 6 hrs.

Since the *γ*-Fe_3_O_4_ phase is more active rather than *α*-Fe_2_O_3_ for photo-Fenton catalysis, the hrGO/*α*-Fe NBhs were reducedinto *γ* phase by N_2_ and H_2_ gas mixture treatment at 600 °C. The reduction period of the hrGO/*α*-Fe NBhs varied by 15 min, 1 h, and 2 h to study the effect of reduction degree on the catalytic activity as shown in Fig. 6. Without photocatalysts, photolysis and self-photosensitization of MB resulted in proportional to degradation rates with zero-order kinetics, showing 40% MB degradation at 1.0 M H_2_ O_2_ loading for 6 hrs (Fig. 6a). At hrGO/*α*-Fe NBhs with identical H_2_ O_2_ concentrations, it showed slightly enhanced photo-degradation efficiency but it was negligible (data not shown here). However, 15 min-H_2_ gas reduced hrGO/*γ*-Fe NBhs led to markedly improvement of MB degradation efficiencies (Fig. 6b). As increase in H_2_ reduction time in hrGO/*γ*-Fe NBhs, photo-Fenton activities of hrGO/*γ*-Fe NBhs for MB degradation were drastically increased at pH 5.8 (Fig. 6c,d). In 1 h-H_2_ reduced cases; 10 mg/L of MB was decolorized with 1.0 M H_2_O_2_ concentration for 6 hrs-photo-Fenton reaction. Particularly, 2 h-H_2_ reduced hrGO/*γ*-Fe NBhs, it exhibited 0.207 (r^2^ = 0.959), 0.370 (r^2^ = 0.954), and 0.721 (r^2^ = 0.904) h^*−*1^ of pseudo-first order rate constants for 0.1, 0.5, and 1.0 M H_2_ O_2_ concentrations, respectively. Importantly, after 10-runs recycles of 2 h-H_2_ reduced hrGO/*γ*-Fe NBhs, the MB degradation efficiencies were a bit decreased, indicating that the 3D hierarchical architecture was retained without deactivating of Fe(0)/Fe_3_O_4_/Fe_2_O_3_ iron oxides. The photo-Fenton mechanism for H_2_O_2_ decomposition, accelerated by UV irradiation, may be due to electron donation from Fe(0) to Fe(III) in Fe_3_O_4_ or Fe_2_O_3_, maintaining persistently Fe(II) ions, which is a predominant H_2_O_2_ decomposition catalyst in Fenton reaction to produce ·OH free radicals. Thus, this non-selective and strong ·OH free radicals become harmless by degradation of MB molecules even at (near) neutral pH. The enhanced photocatalytic performances of the hrGO/*γ*-Fe NBhs were associated with the uniform distribution of discrete *γ*-Fe_3_O_4_ NBs on the 3D hierarchical rGO architecture.

## Discussion

In this study, we have demonstrated the phase controlled solution synthesis of hrGO/*α*-Fe and hrGO/*γ*-Fe NBhs, where their functionalities are controlled for applications into energy storage and photocatalysis. The discrete *α*-Fe_2_O_3_ NBs were nucleated and grown onto the hrGO surface via a one-pot hydrothermal process and uniformly distributed to inhibit the restacking of rGO layers and to maximize their functionalities. Along with the observation of phase transition from *α*- to *γ*-phase, the morphology, chemical structure and composition, and textural property of the hrGO/*α*-Fe NBhs were comprehensively characterized. In order to demonstrate the superiority of the hrGO/Fe NBhs, we applied them into LIB anodes and photo-Fenton catalysis. Despite the low coulombic efficiency and initial capacity, the hrGO/*α*-Fe NBhs showed better rate and cyclic performances than those of commercial *α*-Fe_2_O_3_ due to the electronic conductivity, macroporosity, and buffering effect of the 3D hrGO architecture. Moreover, the catalytic activity and kinetics of hrGO/*γ*-Fe NBhs are enhanced for photo-Fenton reaction because of the uniform distribution of discrete *γ*-Fe_3_O_4_ NBs on the 3D hierarchical rGO architecture. These results provide a chemical strategy for the design of 3D graphene/metal oxide nanocomposite materials that can achieve maximum and multiple functionalities for wide range of emerging applications.

## Methods

### One-pot synthesis of the hrGO/*α*-Fe and hrGO/*γ*-Fe NBhs

The hrGO were synthesized following the previous method^33^,^34^. FeSO_4_ ·7H_2_ O precursor of 1.5 mmol was added into 2 mg/mL of an aqueous graphene oxide (GO) dispersion. The resultant dispersion was sealed in a Teflon-lined autoclave and maintained at 180 °C for 12 hour. Then the autoclave was naturally cooled to room temperature. After gel formation, wet-gel was taken out, washed until pH 7, and freeze-dried into the hrGO/*α*-Fe NBhs. In order to synthesize the hrGO/*γ*-Fe NBhs, the hrGO/*α*-Fe NBhs were reduced by 100 cc/min flowing with H_2_ and N_2_ gas mixture (5 vol%: 95 vol% at 600 °C in the horizontal tube furnace according to the reduction period of 15 min, 1 h, and 2 h.

### Characterization

All samples were analyzed by field emission scanning electron microscopy (FE-SEM, LEO SUPRA 55, 10 kV), corrected scanning transmission electron microscopy (Cs-TEM, JEM-ARM 200F). X-ray diffraction (XRD, D8 Advance) patterns were collected using a General Area Detector Diffraction System (GADDS) (*λ* = 1.5406 *Å*) at 2*θ* between 5° and 90°. X-ray photoelectron spectroscoty (XPS, AXIS Ultra DLD) was used to determine the chemical composition and structure of all samples. Thermogravimetric analysis (TGA) was carried out using a Q5000IR (TA instruments) under air flow (100 ml/min) with a ramp rate of 10 °C/min. Raman spectra were recorded from 100 to 2500 cm^*−*1^ at room temperature using a Raman spectroscopy (RENISHAW inVia Raman Microscope, 785 nm) equipped with a ×100 objective was used. The specific area and pore size distribution were obtained using a Brunauer-Emmett-Teller apparatus (BET, BELSORP-miniII).

### Application into lithium ion battery anode

The battery performances of all samples were evaluated using a Wonatech automatic battery cycler in a CR2016 type coin cell. The electrodes were fabricated by preparing a slurry of 80 wt% active material and 20 wt% polyvinylidene fluoride binder in N-methyl-2-pyrrolidone. The specific capacity was measured based on the mass of active material. The coin cells were assembled by employing a composite electrode with metallic lithium foil and 1M LiPF_6_ (Aldrich 99.99%) dissolved in a solution of ethylene carbonate/dimethyl carbonate/diethyl carbonate (1:2:1 v/v) as an electrolyte in a glove box filled with argon. The cell was galvanostatically cycled between 0.01 and 3.0 V vs. Li/Li^+^ at various specific currents.

### Application into photo-Fenton catalysis

After preparation of methylene blue stock solution (100 mg/L, MB, Sigma-Aldrich, USA) as a cationic model dye, it was diluted with double distilled water (DI water, >18 M*ω* resistance) to 10 mg/mL of 100 mL MB solution in 15 cm-diameter glass dish. UV lamp (VL-6.0LC, 6W, 365 nm wavelenght, France) was utilized at fixed on the hanger with 10 cm height from the glass dish in the desk (0.034 W/cm), offering magnetic stirring at 120 rpm. Hydrogen peroxide (H_2_O_2_, 35%, Junsei, Japan) was employed. 0.01 g of photo-Fenton catalyst was loaded and was regularly sampled with 300 *μ* L at 0, 0.5, 1.0, 2.0, 3.0, 4.0, 5.0, and 6.0 h, and centrifuged samples at 12,000 rpm and 10 min were separated. 100 *μ* L of supernatant was measured at 664 nm wavelength by multi microplate reader (Synergy H-1M, Biotek) automatically. The experiments were conducted at least three times and the resultant data were averaged. For the recycle experiments of photo-Fenton catalyst, after 6 hrs MB decolorization, the MB solution containing catalysts was centrifuged at 6,000 rpm and 15 min, and then remove the supernatant. Finally the catalyst was re-used, additionally along with the preparation of next identical MB solution.

## Additional Information

**How to cite this article**: Yun, S. *et al.* Phase-Controlled Iron Oxide Nanobox Deposited on Hierarchically Structured Graphene Networks for Lithium Ion Storage and Photocatalysis. *Sci. Rep.* **6**, 19959; doi: 10.1038/srep19959 (2016).

## Supplementary Material

Supplementary Information
