# Supplementary material for: Phase-Controlled Iron Oxide Nanobox Deposited on Hierarchically Structured Graphene Networks for Lithium Ion Storage and Photocatalysis
Source: Sci Rep. 2016 Jan 29;6:19959. doi: 10.1038/srep19959 (PMC4731794; doi:10.1038/srep19959)
Supplement: Supplementary Information [file srep19959-s1.pdf]

Supporting information

Phase-Controlled Iron Oxide Nanobox Deposited on Hierarchically Structured Graphene Networks for Lithium Ion Storage and Photocatalysis

Yun Sol[1], Young-Chul Lee[2], Ho Seok Park[1,*]

[1]*School of Chemical Engineering, College of Engineering, Sungkunkwan University, 2066, Seobu-ro, Jangan-gu, Suwon-si, Gyeonggi-do 440-746, Republic of Korea*

[2]*Department of BioNano Technology, Gachon University, 1342 Seongnamdaero, Sujeong-gu, Seongnam-si, Gyeonggi-do 461-701, Republic of Korea.*

* Corresponding authors. Tel: +82-31-299-4715, E-mail: phs0727@skku.edu (Ho Seok Park)

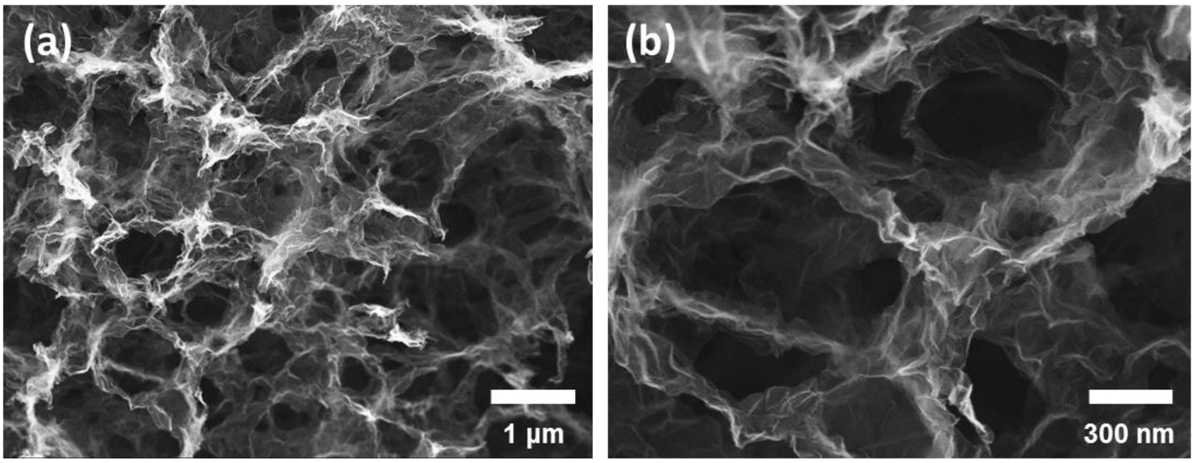

Figure S1. Low and high magnificent SEM of (a) and (b) of hrGO.

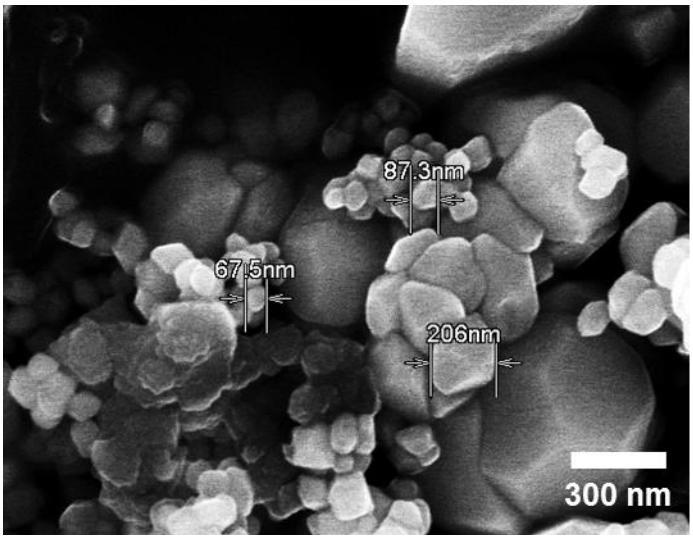

Figure S2. The SEM image of synthesized non-uniform α-Fe_2O_3 particles without rGO under the same synthetic condition as the hrGO/α-Fe NBhs.

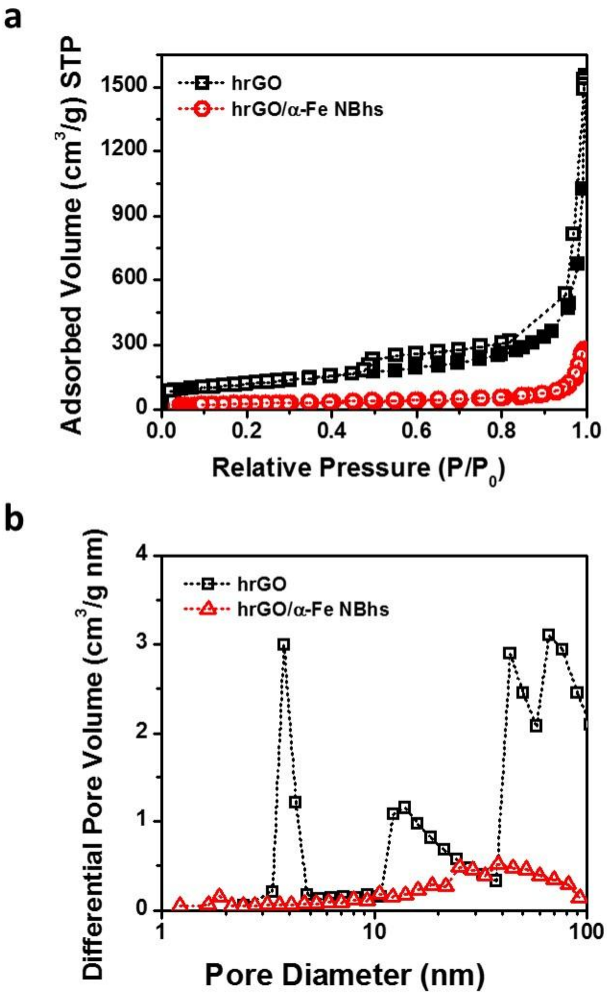

Figure S3. (a) Nitrogen adsorption-desorption isotherm and (b) BJH pore distribution curves of the hrGO and hrGO/α-Fe NBhs.

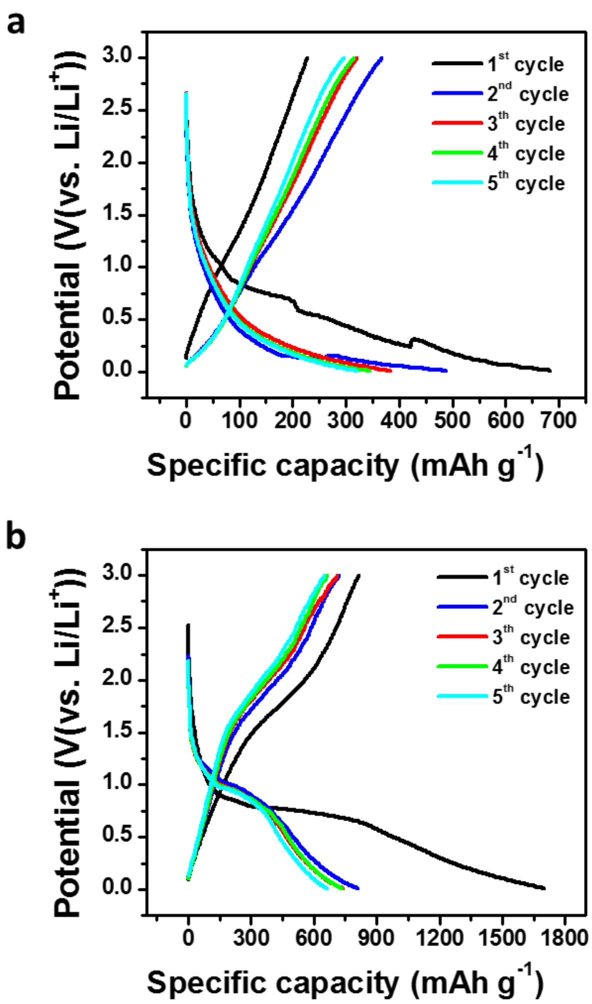

Figure S4. Galvanostatic charge and discharge voltage profiles of (a) hrGO and (b) commercial rGO/α-Fe at a current density of 50 mA/g.

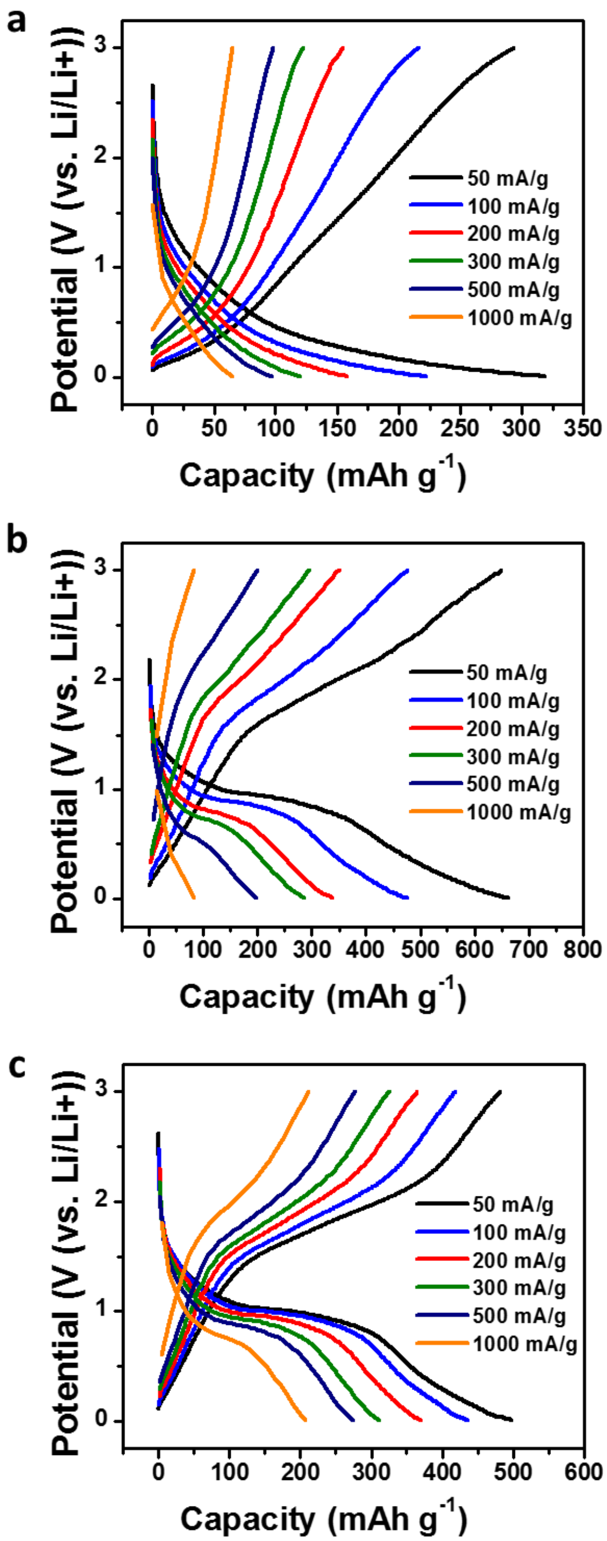

Figure S5. Galvanostatic charge and discharge voltage profiles of (a) hrGO, (b) commercial rGO/α-Fe and (c) hrGO/α-Fe NBhs at various rates from 50 mA/g to 1000 mA/g.

Table S1. The specific capacity of hrGO/α-Fe NBhs based on mass of active material and total.

Specific current density (mA/g)	Specific capacity based on mass of active material (mAh/g)	Specific capacity based on total mass (mAh/g)
50	497.7	398.2
100	434.7	347.7
200	370.0	296.0
300	311.4	249.1
500	274.2	219.4
1000	210.3	168.3

The bulk density of 38.22 mg/cm^3 was obtained by measuring the weight and volume of hrGO/α-Fe NBhs. The loading density '6.34 mg/cm^2' was calculated using weight and area after fabricating the electrode.
